# Subsyndromal symptoms in bipolar disorder

**DOI:** 10.1192/j.eurpsy.2021.541

**Published:** 2021-08-13

**Authors:** A. Mellouli, R. Feki, S. Omri, N. Smaoui, M. Maalej Bouali, N. Charfi, J. Ben Thabet, L. Zouari, M. Maalej

**Affiliations:** 1 Psychiatry C Department, Hedi chaker university hospital, Sfax, Tunisia; 2 Psychiatry C Department, Hedi chaker university hospital, sfax, Tunisia

**Keywords:** bipolar disorder, disease residual minimal, depressive symptoms, manía

## Abstract

**Introduction:**

The inter-critical phase in bipolar disorder may contain symptoms that do not meet the diagnostic criteria for a thymic episode. According to studies, these symptoms are common and usually associated with impaired psychosocial and family functioning.

**Objectives:**

Study the subsyndromal symptoms in remitted patients with bipolar disorder, and their functioning repercussions.

**Methods:**

We conducted a cross-sectional, descriptive and analytical study, in the outpatient psychiatry department of the University Hospital in Sfax (Tunisia) among 30 remitted patients with bipolar disorder. We used: the Montgomery And Asberg Depression Rating Scale (MADRS), the Angst Hypomania Questionnaire and the FAST test to assess functioning levels.

**Results:**

The average age of our population was 44.37±15.45 years with a sex ratio (M/F) =0.66. Most of them lived in urban areas (60%) and half of them did not go beyond the primary school level. Most did not have a constant job (76.6%). The average number of previous thymic episodes was 2±1.33 times/year. A quarter of the patients (26.6%) had hypomanic symptoms in the intercritical phase and 20% had depressive symptoms. Hypomanic symptoms were correlated with tobacco use (p=0.035). Depressive symptoms weremore frequent in men (p=0.074). Functioninglevel was lower in subjects living in rural areas (p=0.065).

**Conclusions:**

Our study suggests that residual symptoms were frequent with a significant functional impact. As a result, their identification and management are highly essential to improve the overall functioning of patients with bipolar disorder.

